# Supplementary material for: Light-driven dandelion-inspired microfliers
Source: Nat Commun. 2023 May 26;14:3036. doi: 10.1038/s41467-023-38792-z (PMC10219969; doi:10.1038/s41467-023-38792-z)
Supplement: Supplementary file 1 — Supplementary Information [file 41467_2023_38792_MOESM1_ESM.pdf]

Supplementary Information

Light-Driven Dandelion-Inspired Microfliers

Yuanhao Chen[1], Cristian Valenzuela[1], Xuan Zhang[1], Xiao Yang[1],

Ling Wang[1,2*] and Wei Feng[1,2*]

[1]School of Materials Science and Engineering, Tianjin University, Tianjin 300350, China; [*]Email: lwang17@tju.edu.cn (L.W.); weifeng@tju.edu.cn (W.F.)
[2]Tianjin Key Laboratory of Composite and Functional Materials, Tianjin 300350, China.

This PDF file includes:

Contents

1. Synthesis and Properties of Bimorph Soft Actuators 1
    1.1 Chemicals and Materials 1
    1.2 Synthesis and characterizations of surface-functionalized ultrasmall AuNRs 1
    1.3 FDTD simulation 2
    1.4 Fabrication of bimorph soft actuators 2
    1.5 COMSOL simulation 3
    1.6 Actuation performance of bimorph soft actuators 4
2. Fabrication and Properties of Dandelion-Inspired Artificial Microfliers 4
    2.1 Fabrication of artificial microfliers 4
    2.2 Light-controlled falling velocity of artificial microfliers 5
    2.3 Light-fueled mid-air flight of artificial microfliers 5
3. Flow Visualization Experiments 6

Other supplementary materials for this manuscript include the following:

Supplementary Movies 1 to 7

1. Synthesis and Properties of Bimorph Soft Actuators

1.1 Chemicals and Materials

All chemicals and materials in this study were used as received: hydroquinone (Sigma-Aldrich), cetyltrimethylammonium bromide (CTAB, Sigma-Aldrich), gold(III) chloride hydrate ($HAuCl_4$, Sigma-Aldrich), sodium borohydride ($NaBH_4$, Sigma-Aldrich), silver nitrate ($AgNO_3$, Sigma-Aldrich), hydrochloric acid (HCl, Sigma-Aldrich), methoxyl polyethylene glycol thiol (PG1-TH-5k, Nanocs), poly(allylamine hydrochloride) (PAH, Aldrich), poly(4-styrenesulfonic acid) (PSS, Aldrich), Sodium chloride (NaCl, Aldrich), Polyimide (PI) film (ZhongXiang, YangZhou), Low-density polyethylene (LDPE) film (FuErFa, ChangZhou), waterborne acrylic adhesive (JiTianHuaGong, ShenZhen), polyacrylonitrile fiber (TianYi, ChangZhou), glass fibers (Taishan Fiberglass, Shandong-China; diameter of 25 μm, and density of 2.4 g cm^{-3}).

1.2 Synthesis and characterizations of surface-functionalized ultrasmall AuNRs

Ultrasmall AuNRs were synthetized by seedless growth method[1] (**Supplementary Fig. 1**). Firstly, 10 mL of CTAB(aq) (0.1M) and 400 mL of $HAuCl_4$(aq) (0.01M) were mixed 90 mL $AgNO_3$(aq) (0.01M). Then, 20 mL of HCl (1M) was added to the growth solution to adjust the pH. The color of the growth solution became transparent from orange color after adding hydroquinone (0.1M) under gentle mixing. Then, 20 mL of freshly prepared ice cold $NaBH_4$(aq) solution was added quickly by using a transfer liquid gun under vigorous stirring. Finally, the mixing solution was conserved at the temperature of 30°C and aged for 10 hours. The AuNRs solution was centrifuged at a rotational speed of 16000 rpm for 30 minutes twice. To obtain negatively charged ultrasmall AuNRs inks, we used mPEG-thiol to replace the CTAB on the surface of ultrasmall AuNRs by ligand exchange[2,3] (**Supplementary Fig. 1**). Briefly, after twice centrifugation, the CTAB-AuNRs dispersion was mixed with 10 mL methoxyl polyethylene glycol thiol (mPEG-SH) (PG1-TH-5k, Nanocs) under vigorous stirring and sonicated for 5 minutes. The mixture was kept still for at least 12 h at room temperature. Then, the mPEG-AuNRs solution was centrifuged at a rotational speed of 8000 rpm to remove excess of mPEG-SH. Finally, AuNR inks were prepared by doping into a waterborne acrylic adhesive.

Ultrasmall AuNRs were characterized with transmission electron microscopy (TEM) imaging (JEM, 2100F, Japan). The size distribution of the ultrasmall AuNRs is shown in **Supplementary Fig. 2**, and the average size 22 ± 5 nm in length and 5.4 ± 2 nm in width. Extinction spectra of the ultrasmall AuNRs solution and mPEG-functionalized ultrasmall AuNRs solution were collected from a cuvette in a UV-Vis-NIR spectrophotometer (Shimadzu, UV-3600 plus, Japan) at room temperature. The zeta potential of the ultrasmall AuNRs was measured with Zetasizer (Malvern, Nano ZS, Britain) at room

temperature. The surface-functionalized ultrasmall AuNRs show significant positive zeta potentials, due to the positively charged amine group in CTAB. After replacing CTAB with mPEG, both solutions show negative zeta potentials because of the methoxy group in the mPEG molecules on the AuNR surface. The FT-IR spectrum of ultrasmall AuNRs was measured with Fourier transform infrared spectroscopy (Thermo, Nicolet5700, America) (**Supplementary Fig. 3**).

1.3 FDTD simulation

The software FDTD Solutions 8.15 was used to simulate the absorption and scattering properties of AuNRs. A built-in model of the software was chosen as the model of the ultrasmall AuNRs. The longitudinal direction of the AuNRs was parallel to the x-axis, and the experimental data of Johnson and Christy were used as the dielectric function of the gold. Taking into consideration of the AuNRs' symmetry, the boundary of AuNRs was set as anti-symmetric condition in the x-min axis and symmetric in the y-min axis. The meshes of AuNRs and their surrounding medium were set as 2 nm and 0.25 nm for large and small AuNRs, respectively. The total-field scattered field source (from 400 nm to 1200 nm) was along the z-axis and polarized parallel to the x-axis. The refractive index of the surrounding medium was set to be 1.33, the refractive index of water.

To calculate the light scattering rate of ultrasmall AuNRs, we controlled the aspect ratio of the ultrasmall AuNRs and used software to simulate the extinction, absorption, and scattering spectra of ultrasmall AuNRs with different sizes. The light absorbance rate of ultrasmall AuNRs is negatively correlated with the size of ultrasmall AuNRs, which means that theoretical absorbance fractions will increase as the size of the ultrasmall AuNRs decreases. To study their optical properties theoretically, we monitored the electric field around ultrasmall AuNRs with the FDTD solution. The color bars represent the ratio between the enhanced electric field and the incident electric field (no unit). It could be enhanced up to 60 times in the vicinity of ultrasmall AuNRs (**Supplementary Fig. 4**).

1.4 Fabrication of bimorph soft actuators

Briefly, a PI film is positively charged via layer-by-layer self-assembly coating using poly(allylamine hydrochloride) and poly(4-styrenesulfonic acid) polyelectrolyte layers[2,3], and subsequently electrostatically laminated with a negatively charged LDPE film evenly spin-coated with an appropriate amount of surface-modified ultrasmall AuNRs. To obtain a positively charged surface, the PI film was firstly dipped into the poly(allylamine hydrochloride) (PAH, Aldrich) solution for at least 5 min, then rinsed with deionized water. Immediately thereafter, the PI film was immersed in the poly(4-styrenesulfonic acid) (PSS, Aldrich) solution for 5 minutes and rinsed in deionized water, so constituting the 1st cycle of the assembly process. At least five cycles were

deposited before the last PAH layer deposition. Next, LDPE film was treated by a plasma cleaner machine (PDC-32G-2, China), then spinning coating with ultrasmall AuNRs inks to form negatively charged LDPE film, which was covered with positively charged PI film to construct a bimorph soft actuator (**Supplementary Fig. 5**).

The contact angle of the PI film before and after modification was obtained by using a sessile drop method (EasyDrop FM40; Kruss, Germany), and it was recorded and analyzed by a built-in camera. The contact angles of polyimide film before and after surface modification were 81.1° and 30.5°, which indicates that the hydrophilicity can be improved after surface modification. Scanning electron microscopy (Hitachi, s4800, Japan) was utilized to measure the surface morphologies of the PI film after deposition of ultrasmall AuNRs, which showed that the thickness of LDPE, PI, and inks were 8 μm, 5 μm, and 400 nm, respectively **(Supplementary Fig. 6)**.

1.5 COMSOL simulation

A finite element analysis model was set up to verify the shape-bending mechanism of the bimorph soft actuator by using COMSOL Multiphysics software 5.4. The model was simplified to a two-dimensional design and some parameters of film are provided in **Supplementary Table 1**. The length of the model was 500 μm and the thicknesses of the two layers are 8 μm and 5 μm, representing the LDPE and PI films, respectively. The “Solid Mechanics” and “Thermal Expansion” physical modules were used to simulate the thermal actuation and study the steady state temperature-induced deformation at a reference temperature of 25 °C. The temperature distribution is essentially uniform because the thickness of the actuator (< 14 μm) is typically less than 1/1285 of the length in the other two dimensions (~18 mm). therefore, the heat conduction inside the actuator is very fast. Based on the bimetal thermostat analysis the deformation radius is[4]:

$$\rho = \frac{(h_1+h_2)(3(1+m)^2+(1+mn)(m^2+\frac{1}{mn}))}{6(\alpha_2-\alpha_1)(1+m)^2}\frac{1}{(T-T_0)} \quad (1)$$

Here,

$$m = \frac{h_1}{h_2},\ n = \frac{E_1}{E_2}$$

Where 1 and 2 represent PI and LDPE, respectively. h_1 and h_2 are the thickness of PI film and LDPE film, respectively, E_1 and E_2 denote their moduli of elasticity, α_1 and α_2 denote their coefficients of expansion, T_0 is the room temperature (25 °C) at which the sample is flat. Once the temperature changed, the force distributions and shape-bending performances of actuators were re-established as shown in **Fig. 2b.** In fact, for a small section of sample, there is the following relationship between the bending angle and the bending radius,

$$\Delta\theta = \frac{\Delta l}{\rho} \quad (2)$$

We simulated and measured the deformation of actuators from ΔT = 0 °C

to 16 °C, and the result showed that the bending angle is positively related to the temperature (**Supplementary Fig. 7)**.

1.6 Actuation performance of bimorph soft actuators

The mechanical property of the film was measured by a mechanical tester (Suns 890, China). we define α as the angle between the longitudinal direction of the film and the axis of the LDPE along the direction of low CTE value. To confirm the anisotropy of LDPE[7-9], we measured the mechanical properties of LDPE films in three directions (α=0°, 45° and 90°). The LDPE films were cut into strips with length of 40 mm and width of 5 mm. We set the clamping distance to 20 mm, the stretching speed to 10 mm min^{-1}, and each sample was tested at least 5 times, and **Supplementary Fig. 8** demonstrates the anisotropy of LDPE layer.

Upon heating, the uneven expansion of the LDPE layer produces a twisting force whose direction closely depends on the cutting angle α (90°, 135°, and 45°), thus leading to shape deformations from an elongated planar shape into a cylindrical and left- or right-twisting shape, respectively. The high sensitivity of our actuators enabled them to feedback to a slight change in temperature, even the surface temperature of the human skin would make them deform rapidly. There was a gap of 8 °C between room temperature (24 °C) and surface temperature of the hand (32 °C) **(Supplementary Fig. 9)**.

The camera (Canon, EOS 6D Mark II, Japan) was utilized to record the responsiveness of actuators after IR light was turned on, and the response time and deformation amplitude were analyzed by Corel Video Studio (Corel, X9, Canada) and Photoshop (Adobe, CC 2019, American). The temperature of bimorph soft actuators under the NIR light irradiation (Changchun New Industries Optoelectronics Technology Co., 0~10 W, wavelength of 808 nm) was measured by infrared camera (IRC, R500Ex-Pro-D, Japan). Under the NIR light, the bimorph soft actuator exhibited a very fast response speed and efficient photothermal effect. At the same time, we found that its surface temperature showed a linear relationship with the light intensity. The maximal bending angle was measured five times and obtained an average value under different light intensities from 10 mW cm^{-2} to 200 mW cm^{-2} **(Supplementary Fig. 10)**.

2. Fabrication and Properties of Dandelion-Inspired Artificial Microfliers

2.1 Fabrication of artificial microfliers

To fabricate a dandelion-inspired artificial microflier, one end of the bimorph soft actuator film (α=90°, 14 mm × 12 mm) is cut evenly along the longitudinal direction to obtain many uniform thin strips (5 mm in length and ~0.7 mm in width), and a tubular bimorph soft actuator is further constructed with the LDPE film as the inner layer and the PI film as the outer layer as schematically shown in the **Supplementary Fig. 13**. The artificial microflier can be obtained after the

thin strips were attached by an appropriate amount of fiberglass strands with a length of 10~20 mm ("pappus"), and the weights of all microfliers are adjusted to about 4.0 mg in the experiments.

2.2 Light-controlled falling velocity of artificial microfliers

To avoid disturbance of surrounding air, all the experiments were conducted in a confined space. At the top of the space, an infrared light was set to control the shape-morphing of artificial microfliers, and the artificial microflier was released from a proper height upon light irradiations. The camera (Canon, EOS 6D Mark II, Japan) was used to record the falling process of the artificial microflier and its falling velocity and opening angle during falling process were analyzed by Corel Video Studio (Corel, X9, Canada) and Photoshop (Adobe, CC 2019, American).

During the free falling progress of the artificial microflier under the light, the air drag is taken into account, so newton's second law for this situation is (**Supplementary Fig. 20**):

$$\sum F = F_{\mathrm{g}} - F_{\mathrm{D}}(t) = m\frac{dv}{dt} \quad (1)$$

Where $F_g = mg$ is the gravitational force, m = 4×10^{-6} kg is the effective mass of the artificial microflier as measured by an electronic balance, g = 9.81 N Kg^{-1} is the constant of acceleration near Earth's surface, and the positive direction of velocity is downward, $F_D(t)$ is the air drag force. The direction of the drag force always opposed the velocity. Thus, the equation expressing this force was given by[5,6],

$$F_{\mathrm{D}}(t) = 0.5C_{\mathrm{D}}\rho A v^2(t) \quad (2)$$

Where C_D is the drag coefficient related to the projected area, ρ is the air density (1.2 kg m^{-3}), A is the projected area, v(t) is the falling velocity, however, according to Newton's second law, the velocity gradually increased during falling until $\sum F = 0$, which is due to we only recorded and analyzed the terminal velocity of the artificial microflier. When $\sum F = 0$, the terminal velocity is given by:

$$v = \left(\frac{2mg}{C_{\mathrm{D}}\rho A}\right)^{1/2} \quad (3)$$

At this point, the drag force is balanced with gravity, and the artificial microflier will fall at a uniform velocity. The schematic diagram of projected areas at different angles ϕ is shown in **Supplementary Fig. 17**, where the ϕ is related to the light intensity. Thus, the falling velocity of the artificial microflier is slower when the light is turned on.

2.3 Light-fueled mid-air flight of artificial microfliers

In the flight experiment, an airflow tunnel was designed to generate updrafts above light source, where a wide-band infrared light lamp (Philips infrared lamp, 0~350 W, wavelength range: 420 nm~1000 nm) and a simulated solar light (Ceaulight lamp, 0 W~300 W, wavelength range: 350 nm~1000 nm) were used

as light source. The artificial microflier was held above the tunnel's outlet with a tweezer, and the light-induced updraft provides the artificial microfliers with lift force. All the experiments were conducted in a confined space to prevent surrounding airflow disturbance. The opening angle of artificial microfliers was obtained and recorded by camera. The light intensity is tested by light power meter (Ceaulight, CEL-NP2000-10).

As a comparison, the artificial microfliers without thin strips could not deform in response to light (**Supplementary Fig. 23**), which explained the importance of projected area. In addition, the air vortex above the artificial microflier and its size was closely related to the angle of the pappus (**Supplementary Fig. 28)**. To analyze the flight mechanism of artificial microflier, we also tested the flight ability of real dandelion, which was placed above the same device and recorded by a camera. Dandelion seeds were collected from a single plant growing at Tianjin university.

The updraft produced by light was the lift force for the flight of the artificial microflier. The updraft velocity v_c in the center of the light tunnel is shown in **Supplementary Fig. 30.** Here, the direction of the drag force was the same as v_c, so the drag force obtained from the updraft was:

$$F_D = 0.5 C_D \rho A v_c^2 \quad (4)$$

According to Newton's second law and the expression (1), if $\sum F < 0$, meaning $mg < F_D$, the artificial microflier would be lifted by the updrafts produced by the light.

3. Flow Visualization Experiments

The flow visualization experiment was implemented in a confined space to prevent surrounding airflow disturbance. A fog machine (Yakay PT-1500) was used to seed the air through the airflow tunnel with a length of 400 mm and diameter of 100 mm, and it was illuminated by using a 10-W laser LDY DualPower 304 (diode-pumped, dual cavity, Q-switched Nd: YLF) with a wavelength of 532 nm. The videos were obtained using Canon EOS 6D Mark II (for low-speed videos) and 10-bit high-speed CMOS cameras with 200 mm Micro-Nikkor lens (for high-speed videos). Particle image velocimetry (PIV) was performed using a 532 nm laser to illuminate air that is seeded with smoke. The TR-PIV (Time-resolved particle image velocimetry) was employed to measure the flow field of the turbulent boundary layer in the x–y plane (135 mm × 70 mm). According to the flow velocity, the high sampling frequency was set to 400 Hz and 4000 instantaneous snapshots of particle images were then captured. There were about 20 particles in each interrogate window (32 × 32 pixels) and the overlap rate was 75%. Universal outlier detection in Dantec Dynamic Studio was used to remove the noise, and the proportion of interpolation vectors was less than 1%.

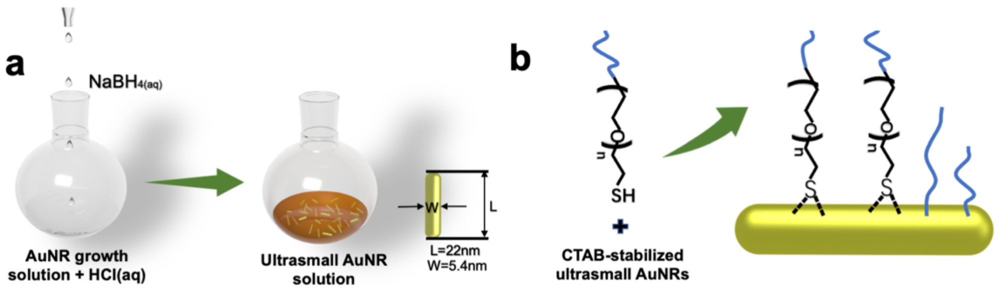

Supplementary Fig. 1. **The synthesis and surface functionalization of ultrasmall AuNRs.** Schematic illustration of **a** seedless growth to produce ultrasmall AuNRs and **b** surface functionalization. CTAB: cetyltrimethylammonium bromide; mPEG-SH: methoxyl polyethylene glycol thiol.

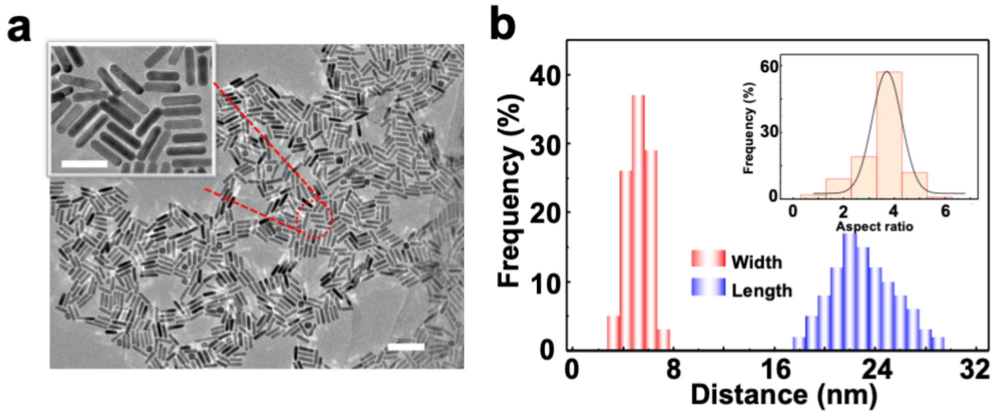

Supplementary Fig. 2. The morphology analysis of ultrasmall AuNRs. a TEM image of ultrasmall AuNRs (scale bar, 50 nm). The inset chart shows the TEM image of a partial enlargement (scale bar, 20 nm). **b** Size distribution of the ultrasmall AuNRs, confirming the ultrasmall AuNRs with dimensions of 22 nm by 5.4 nm. The inner chart is the distribution of the aspect ratio of ultrasmall AuNRs.

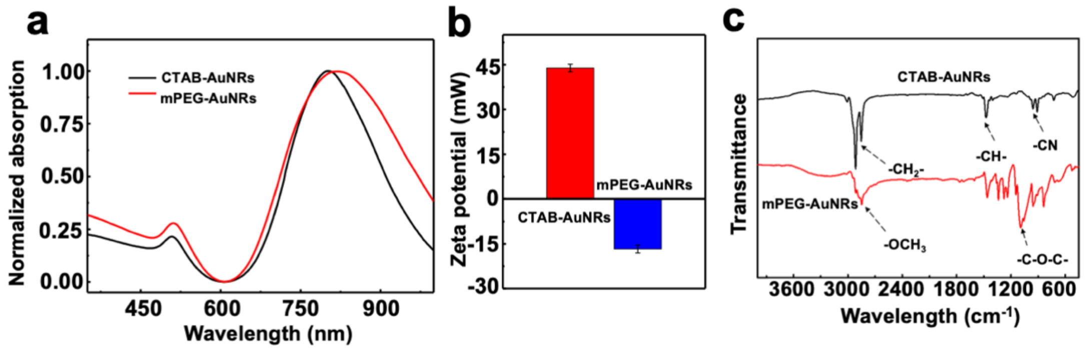

Supplementary Fig. 3. The comparison of **a** the normalized UV-Vis-NIR absorption spectrum, **b** zeta potential, and **c** FT-IR spectrum of ultrasmall AuNRs before and after surface modification.

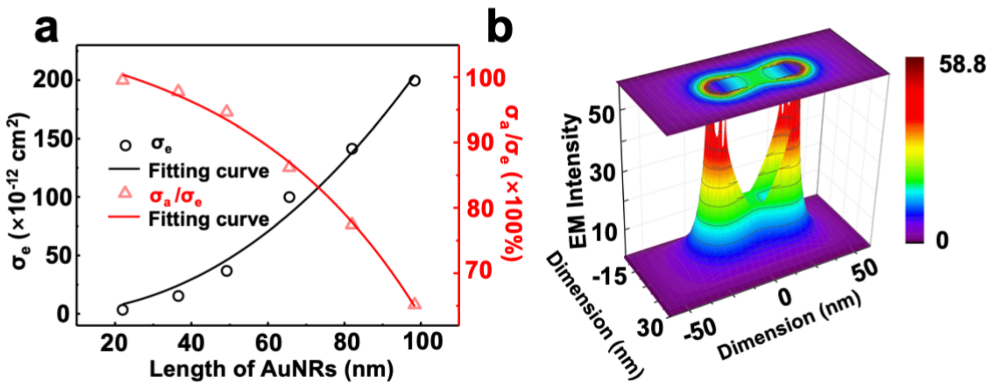

Supplementary Fig. 4. The FDTD simulation of AuNR. a The FDTD simulation calculation of extinction and absorption-to-extinction fractions as the function of the AuNRs' size. **b** The FDTD simulation of electric field enhancement of ultrasmall AuNRs.

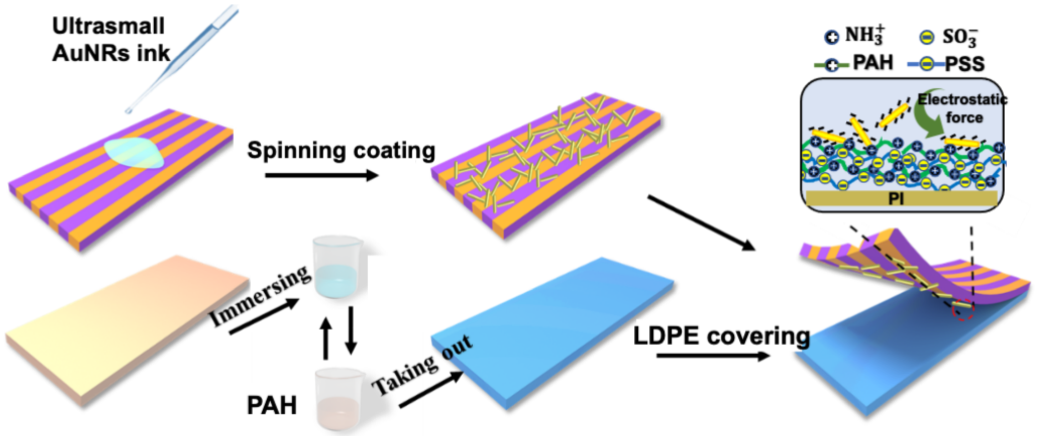

Supplementary Fig. 5. Schematic illustration showing the preparation process of the bimorph soft actuators and the electrostatic forces between PI and LDPE films.

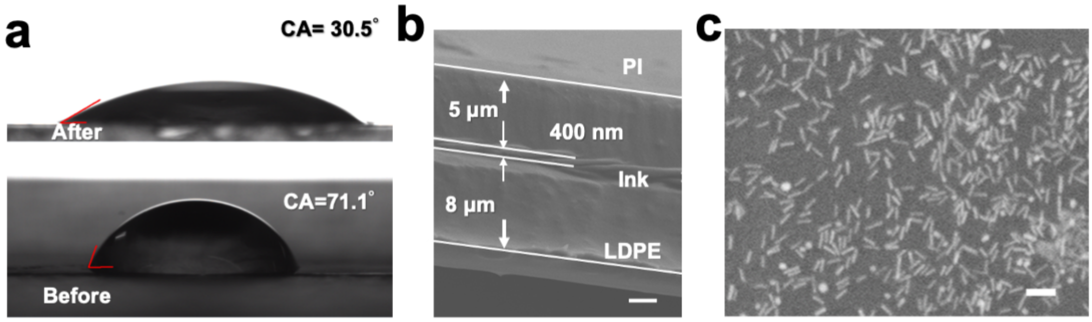

Supplementary Fig. 6. Contact angle and morphology analysis of soft actuators. a Contact angle change before and after surface modification of polyimide film. **b** Cross-sectional SEM image of the bimorph soft actuators (scale bar 2 μm). **c** The distribution of AuNRs on the LDPE film (scale bar 50 nm).

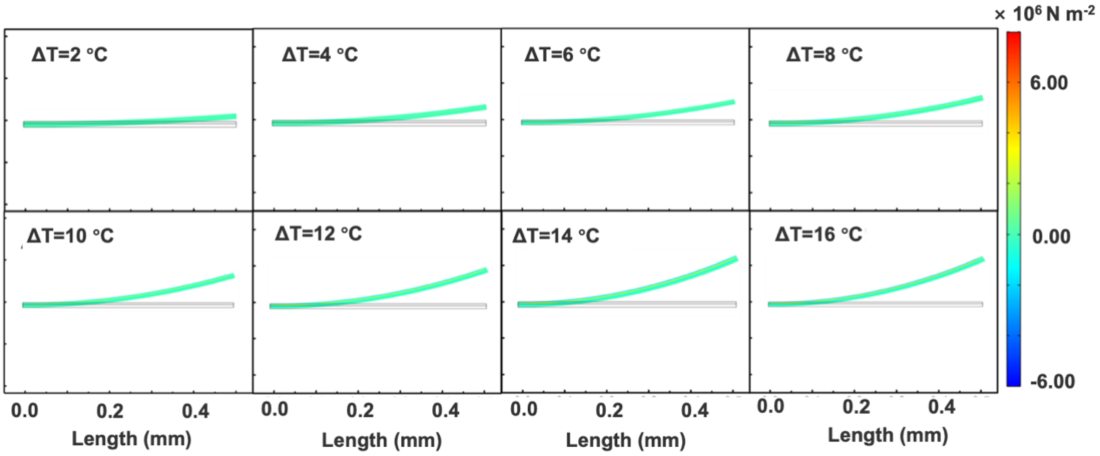

Supplementary Fig. 7. Deformation and shear force of bimorph soft actuators by imposing different temperatures.

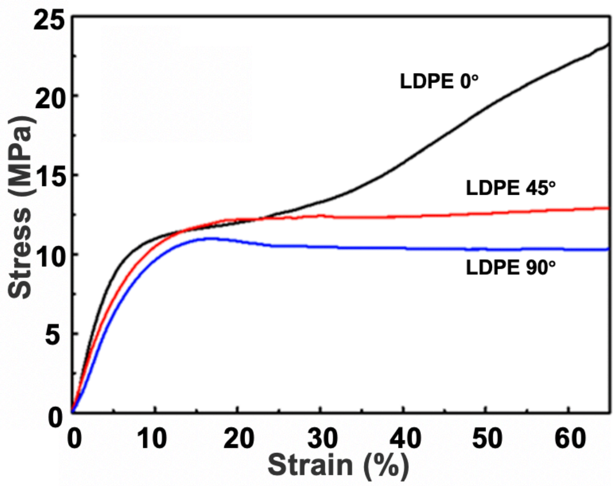

Supplementary Fig. 8. Stress-strain curves for the LDPE in different directions.

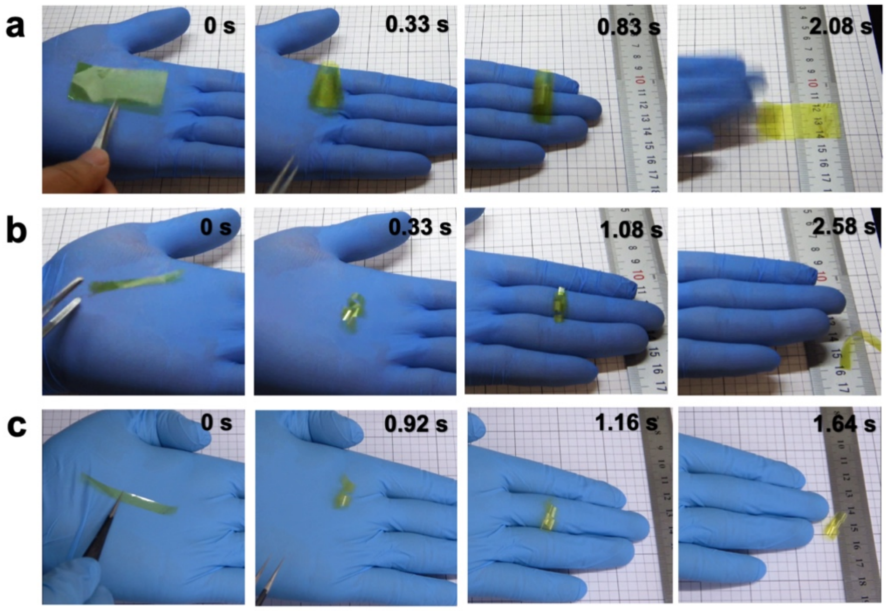

Supplementary Fig. 9. The deformation of soft actuators on the hand. a Photographs of strip A (length of 45 mm, width of 25 mm, and thickness of 13.4 μm) deforming into a

cylinder-like shape and rolling down from the hand. The room temperature was 24 °C and the surface temperature of hand was 32 °C. **b** Photographs of strip B (length of 45 mm, width of 5 mm, and thickness of 13.4 μm) deforming into a left-twisting shape and rolling down from the hand. **c** Photographs of strip C (length of 45 mm, width of 5 mm, and thickness of 13.4 μm) deforming into right-twisting shape and rolling down from the hand.

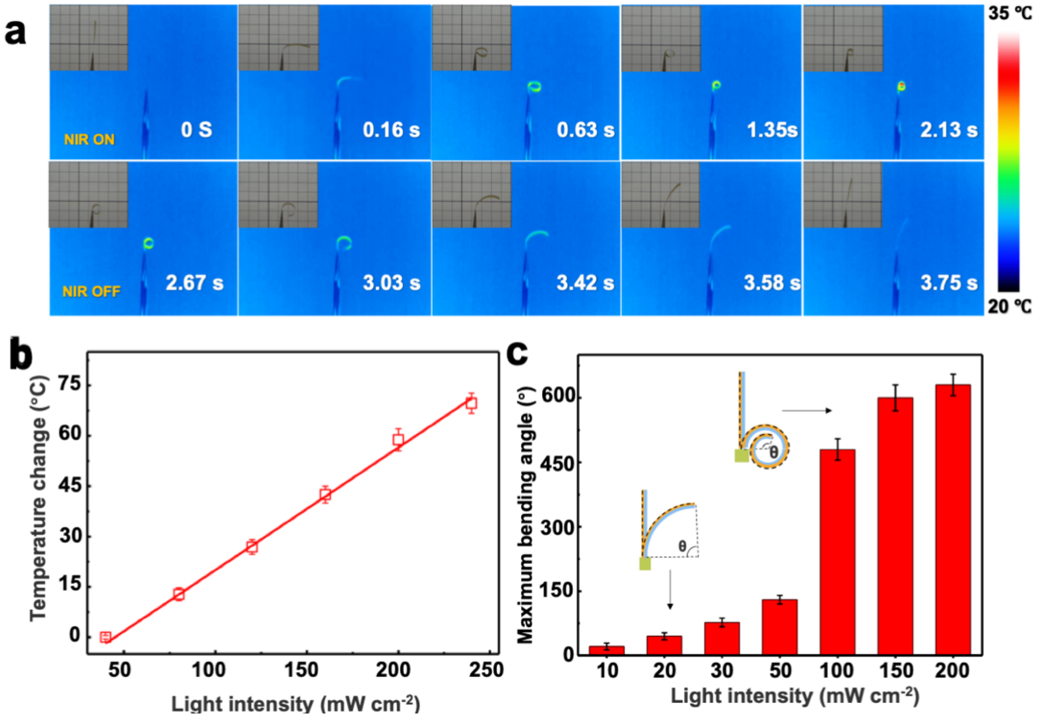

Supplementary Fig. 10. Ultralight and super-sensitive bimorph soft actuator in response to light. a Series of infrared images shows the bending angle and temperature change of bimorph soft actuator when NIR light (808 nm, 150 mW cm^{-2}) is turned on and off (scale bar, 10 mm). The inset chart shows the real-time bending upon NIR light irradiation. **b** The measured maximum temperature for bimorph soft actuator as a function of NIR light intensity. **c** The measured maximum bending angle for bimorph soft actuator as a function of NIR light intensity (808 nm, 10–200 mW cm^{-2}). When the bending angle does not exceed 360°, it is “θ”; When the bending angle exceeds 360°, it is “(θ+360°).

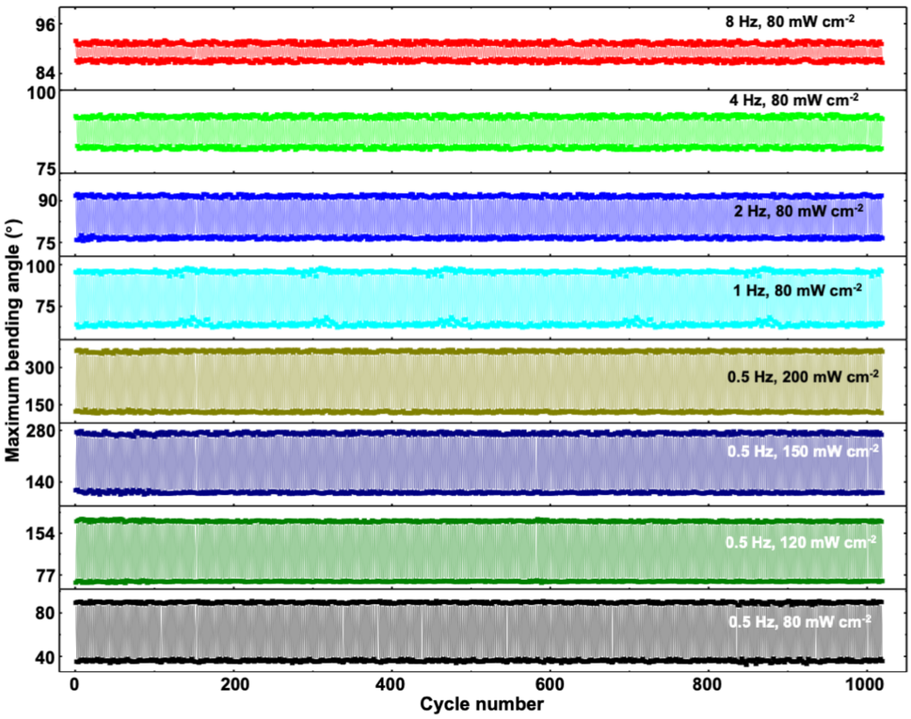

Supplementary Fig. 11. Repeated actuation of soft bimorph actuators under different frequencies and light intensities (NIR, 808 nm). In each cycle, the irradiation time accounts for half of the cycle time.

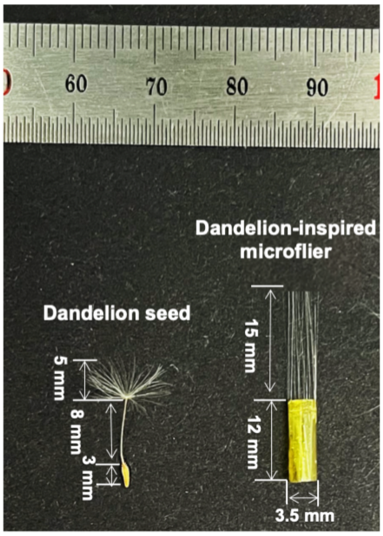

Supplementary Fig. 12. Dimensions comparison of a real dandelion seed and dandelion-inspired microflier. The length of the "pappus" of the microflier can be set to 10 mm, 15 mm, or 20 mm by cutting.

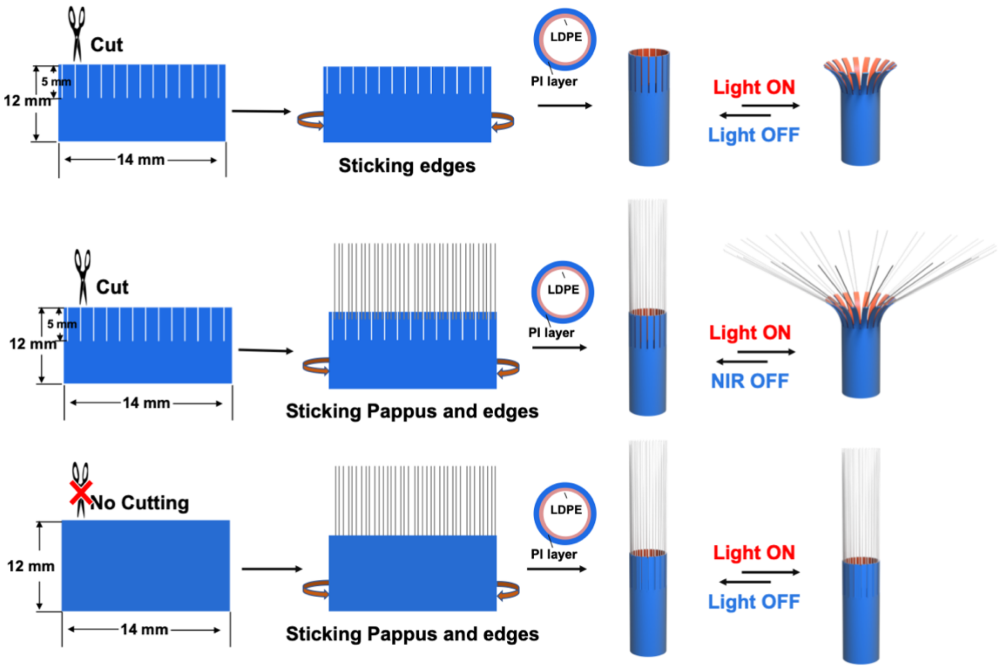

Supplementary Fig. 13. The fabrication process of dandelion-inspired artificial microfliers. a artificial microfliers without pappus, **b** artificial microfliers with pappus, and **c** artificial microfliers showing no response to light.

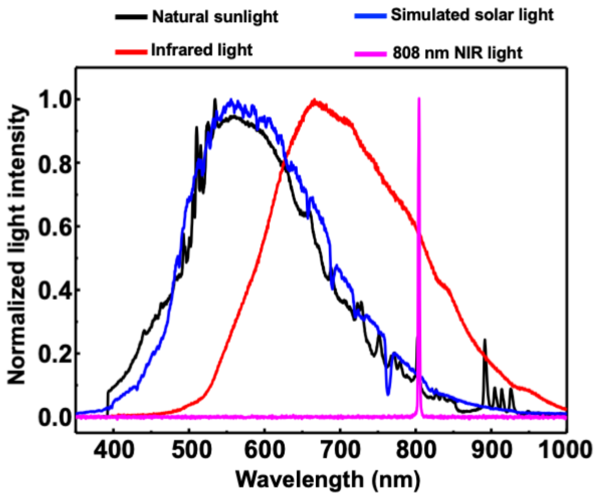

Supplementary Fig. 14. The optical spectra of different light sources used in the experiment: Natural sunlight, simulated solar spectrum (Ceaulight lamp, 0 W~300 W), infrared light (Philips infrared lamp, 0~350 W) and 808 nm NIR light (Changchun New Industries Optoelectronics Technology Co., 0~10 W).

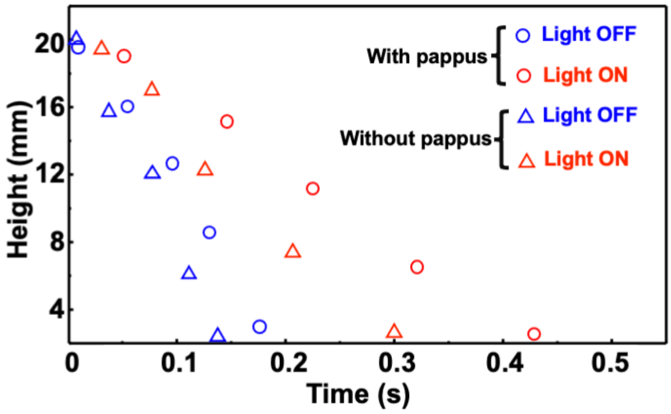

Supplementary Fig. 15. Flight time of dandelion-inspired microfliers with and without "pappus" falling from a fixed height h.

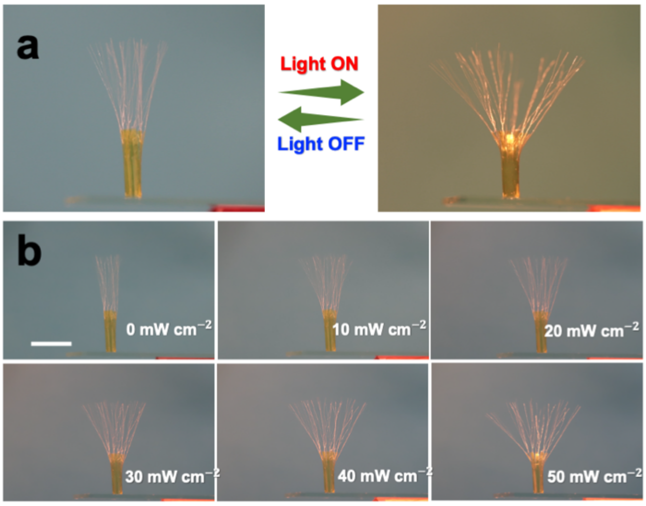

Supplementary Fig. 16. The "pappus" opening of artificial microflier under light irradiation. a The "pappus" opening of artificial microflier in response to light (Philips infrared lamp, 50 mW cm^{-2}, wavelength range: 420 nm~1000 nm). **b** The maximum opening angle of the "pappus" of a microflier under light irradiation with different intensities (scale bar: 10 mm).

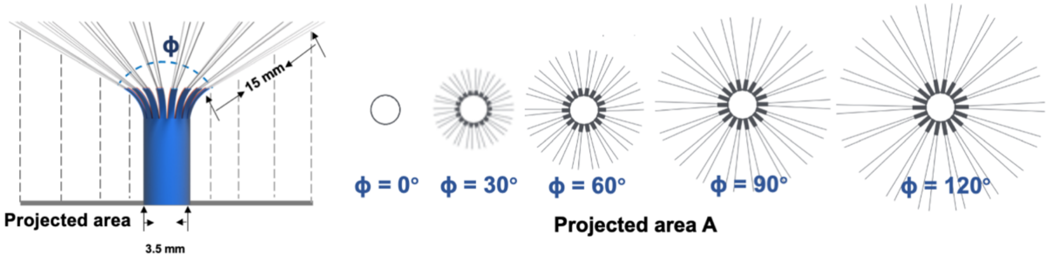

Supplementary Fig. 17. Schematic illustration of artificial microflier's projection area at different opening angles of "pappus".

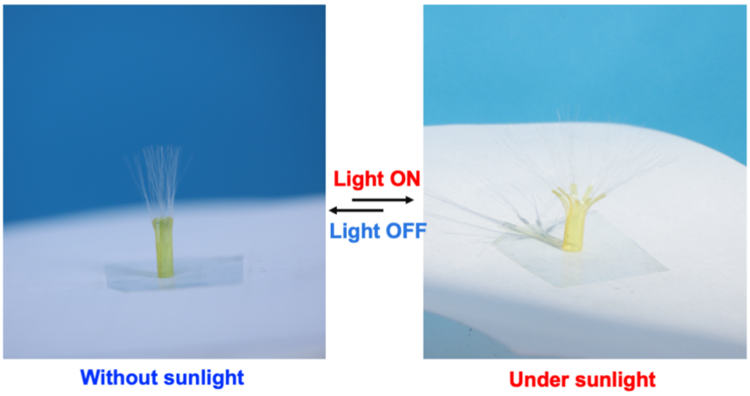

Supplementary Fig. 18. The “pappus” opening of artificial microflier upon being exposed to natural sunlight (~80 mW cm^{-2}) at room temperature.

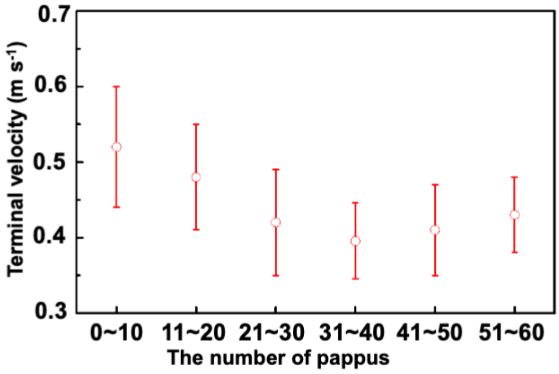

Supplementary Fig. 19. The terminal falling velocity of artificial microflier with different number of fiberglass strands (pappus) under the infrared light (50 mW cm^{-2}).

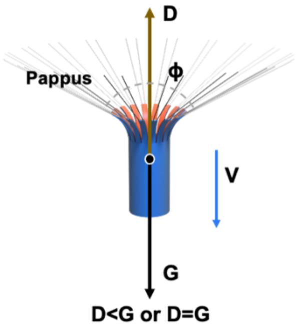

Supplementary Fig. 20. Force analysis of the artificial microflier during falling: the artificial microflier with opening angle of pappus ϕ (the angle between the outermost pappus) has a falling velocity v, which possess the gravity G and the drag force D.

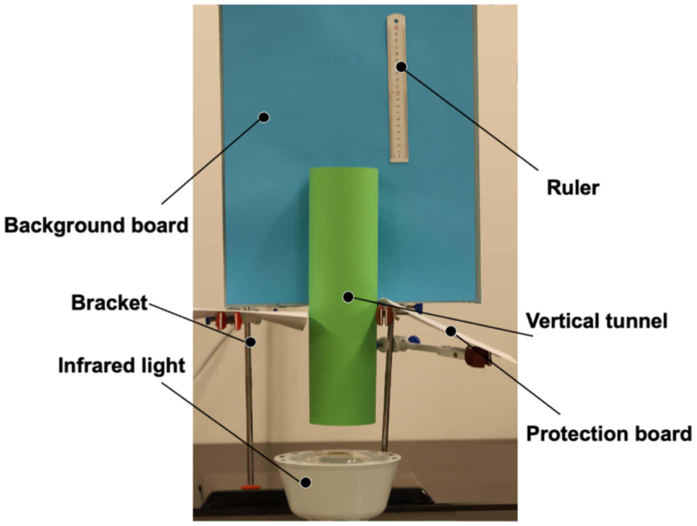

Supplementary Fig. 21. Photograph of the experimental optical setup for the light-driven mid-air flight of artificial microflier.

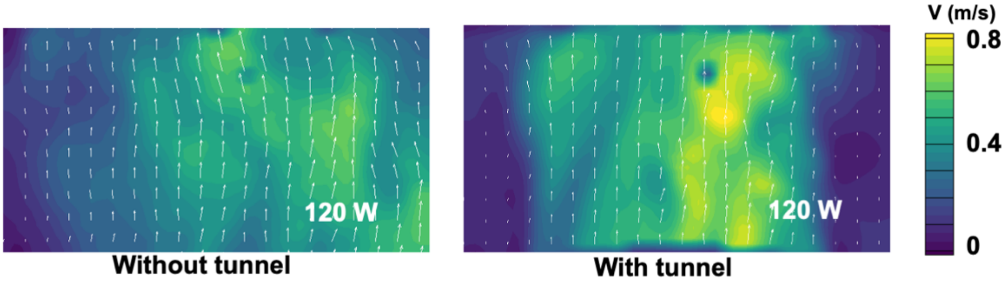

Supplementary Fig. 22. Light-induced updraft distribution with tunnel and without tunnel above the light irradiation (Philips infrared lamp, 120 W).

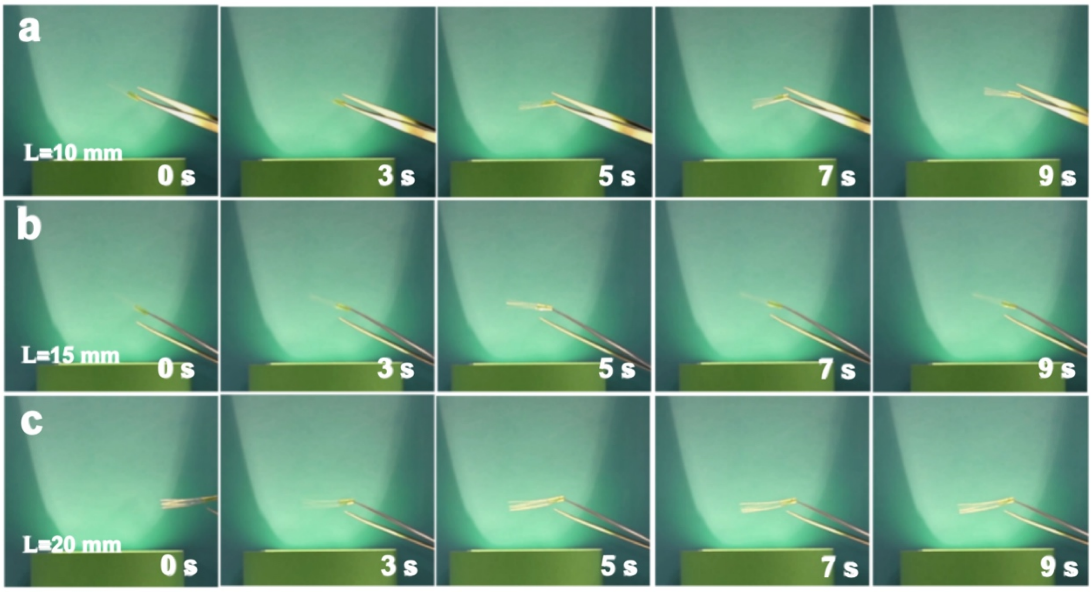

Supplementary Fig. 23. The flight test of artificial microfliers (always kept in a closed state). Photographs show that the artificial microfliers cannot fly although attached to **a** 10 mm, **b** 15 mm, and **c** 20 mm pappus because are kept in a closed state in response to light (Philips infrared lamp, 300 W), which demonstrates the importance of the projected area for the flight of artificial microflier.

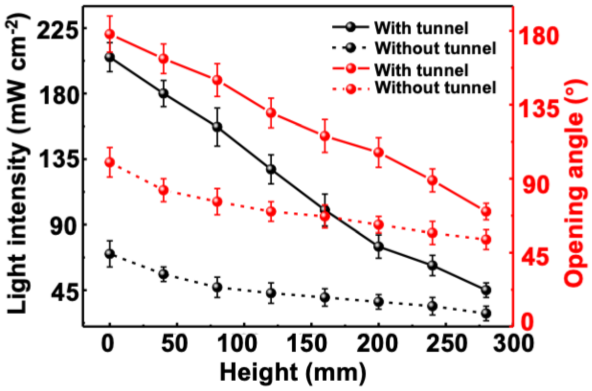

Supplementary Fig. 24. Light intensity and "pappus" opening angle of artificial microfliers with tunnel and without tunnel at different heights (Philips infrared lamp, 300 W).

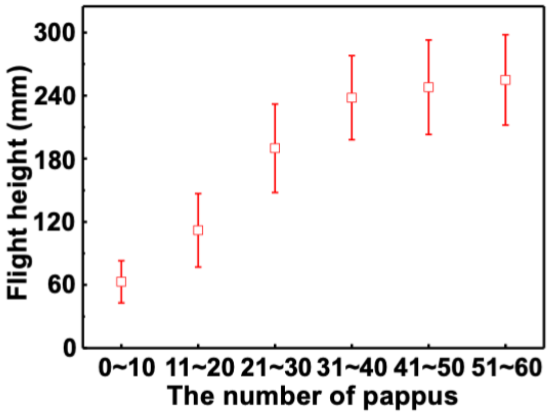

Supplementary Fig. 25. The average flight height of the microfliers with different numbers of fiberglass strands (the length of pappus is fixed at 15 mm) under constant light irradiation (Philips infrared lamp, 300 W).

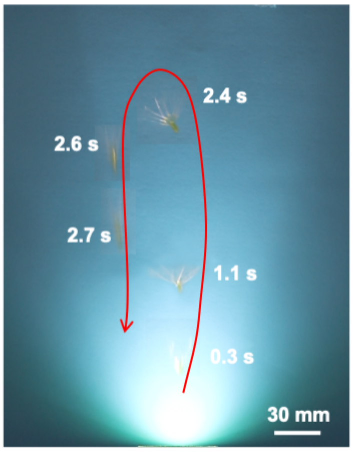

Supplementary Fig. 26. The flight process of as-prepared microfliers above the simulated solar light (Ceaulight lamp, 300 W).

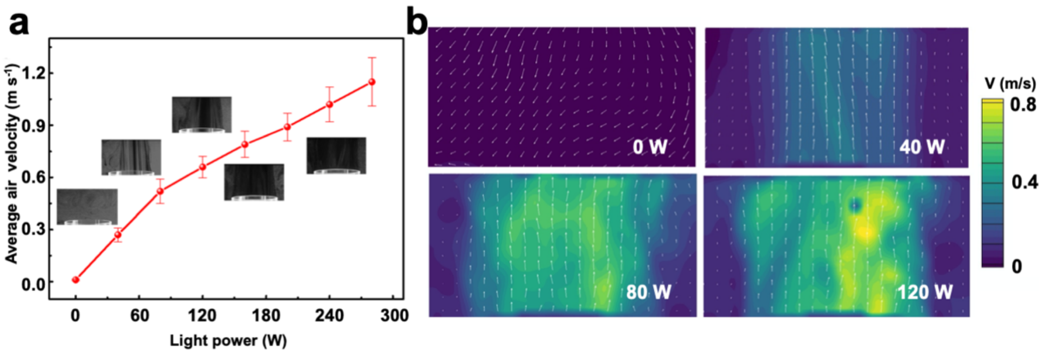

Supplementary Fig. 27. The air velocity test above the optical setup. a The average air velocity at different light powers (Philips infrared lamp) from 0 W to 300 W; The inset figures are the flow visualization captured by camera. The average air velocity is obtained from ten separate tests. **b** The comparison of the airflow velocity in presence of the light when an artificial microflier is passing through the tunnel among different light powers (Philips infrared lamp) of 0 W, 40 W, 80 W, and 120 W.

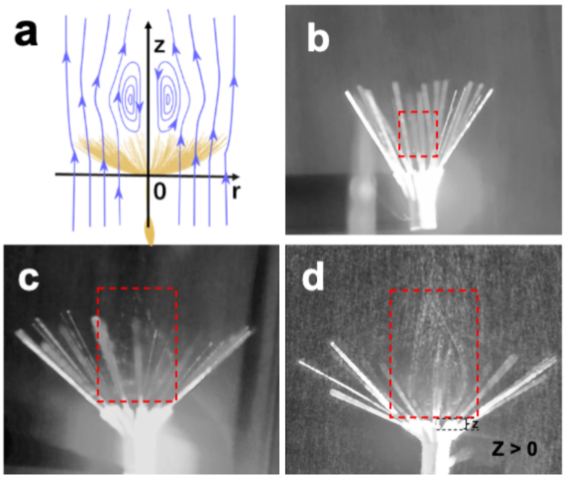

Supplementary Fig. 28. PIV characterization showing air vortex rings above the microfliers. a Schematic illustration of the air distribution above artificial microflier under light irradiation (Philips infrared lamp, 120 W). Stacked images of air vortex rings above the microfliers with different fixed “pappus”opening angles: **b** 60°, **c** 90°, and **d** 120°. The red rectangle is where the air vortex occurs. Z is the distance between artificial microflier and stable vortex rings, here, Z > 0 indicates that the vortex is separated.

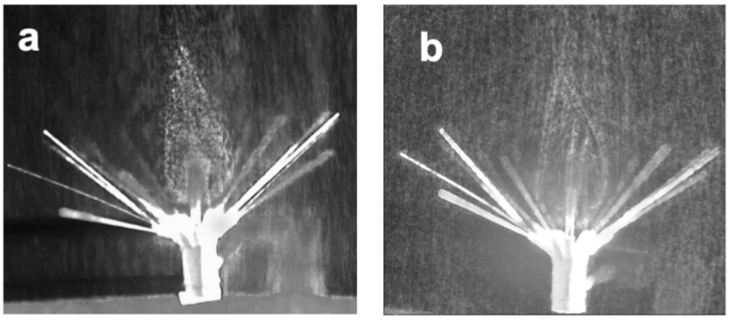

Supplementary Fig. 29. The air vortex rings above the artificial microflier with a fixed “pappus” opening angle of 120° under light irradiation (Philips infrared lamp, 120 W). a Tubular-shaped bimorph soft actuator is blocked. **b** Tubular-shaped bimorph soft

actuator is open.

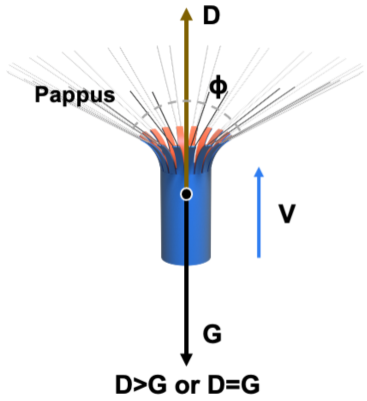

Supplementary Fig. 30. **Force analysis of the artificial microflier during flight.** The artificial microflier with an opening angle of pappus ϕ (the angle between the outermost pappus) has a flight velocity v, which is governed by the gravity G and the drag force D.

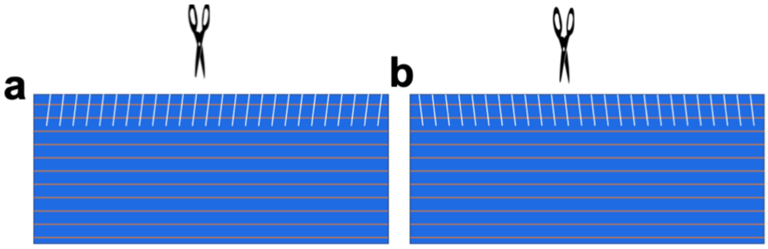

Supplementary Fig. 31. **The cutting process of bimorph soft actuators. a**, **b** Schematic illustration showing the slight angular deviations. The orange line is the direction of the low CTE of LDPE.

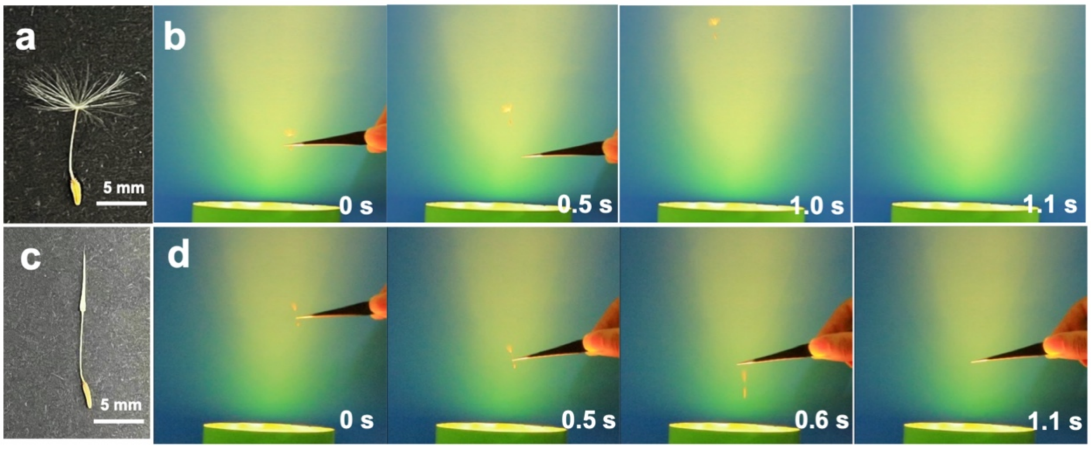

Supplementary Fig. 32. **The light-fueled mid-air flight of a real dandelion seed.** Real dandelion seeds with **a, b** open pappus and **c, d** closed pappus above a light source (Philips infrared lamp, 300 W).

Supplementary Table 1. Physical parameters of LDPE and LDPE.

Property	LDPE[7-9]	PI[10-12]	Unit
Young's modulus	$2.02e^{8}$	$3.2e^{9}$	Pa
Poisson's ratio	0.4	0.34	1
Density	910	1420	Kg m^{-3}
CTE	$280e^{-6}$	$20e^{-6}$	K^{-1}

References:

1. Chen, Y. S. et al. Miniature gold nanorods for photoacoustic molecular imaging in the second near-infrared optical window. *Nat. Nanotechnol.* **14**, 465-472 (2019).
2. Kang, H. et al. Inkjet-printed biofunctional thermo-plasmonic interfaces for patterned neuromodulation. *ACS Nano* **12**, 1128-1138 (2018).
3. Kang, H., Lee, J. W. & Nam, Y. Inkjet-printed multiwavelength thermoplasmonic images for anticounterfeiting applications. *ACS Appl. Mater. Inter.* **10**, 6764-6771(2018).
4. Timoshenko, S. Analysis of bi-metal thermostats. *Josa* **11**, 233-255 (1925).
5. Parker, G. W. Projectile motion with air resistance quadratic in the speed. *Am. J. Phys.* **45**, 606-610 (1977).
6. Greene, D. F. & Johnson, E. A. The aerodynamics of plumed seeds. *Funct. Ecol.* 117-125 (1990).
7. Olmos, D., Martínez, F., González-Gaitano, G. & González-Benito, J. Effect of the presence of silica nanoparticles in the coefficient of thermal expansion of LDPE. *Eur. Polym. J.* **47**, 1495-1502 (2011).
8. Almanza, O., Masso-Moreu, Y., Mills, N. J. & Rodríguez-Pérez, M. A. Thermal expansion coefficient and bulk modulus of polyethylene closed-cell foams. *J. Polym. Sci. B Polym. Phys.* **42**, 3741-3749 (2004).
9. Zhang, X. M., Elkoun, S., Ajji, A. & Huneault, M. A. Oriented structure and anisotropy properties of polymer blown films: HDPE, LLDPE and LDPE. *Polymer*, **45**, 217-229 (2004).
10. Numata, S. & Miwa, T. Thermal expansion coefficients and moduli of uniaxially stretched polyimide films with rigid and flexible molecular chains. *Polymer* **30**, 1170-1174 (1989).
11. Bauer, C. L. & Farris, R. J. Determination of poisson's ratio for polyimide films. *Polym. Eng. Sci.* **29**, 1107-1110 (1989).
12. Felder, R. M., Patton, C. J. & Koros, W. J. Dual-mode sorption and transport of sulfur dioxide in kapton polyimide. *J. Polym. Sci. Pol. Phys.* **19**, 1895-1909 (1981).
