# Supplementary material for: Light-driven dandelion-inspired microfliers
Source: Nat Commun. 2023 May 26;14:3036. doi: 10.1038/s41467-023-38792-z (PMC10219969; doi:10.1038/s41467-023-38792-z)
Supplement: Supplementary file 3 — Description of Additional Supplementary Files [file 41467_2023_38792_MOESM3_ESM.docx]

**Description of Additional Supplementary Files**

**File Name: Supplementary Movie 1
Description:** Shapeprogrammability of bimorph soft actuator films.

**File Name: Supplementary Movie 2
Description:** Light-controlled falling velocity of dandelion-inspired artificial microfliers.

**File Name: Supplementary Movie 3**

**Description:** The light-fueled mid-air flight of dandelion-inspired artificial microfliers.

**File Name: Supplementary Movie 4**

**Description:** Light-driven updraft above a tunnel.

**File Name: Supplementary Movie 5**

**Description:** The separated vortex ring above dandelion-inspired microfliers.

**File Name: Supplementary Movie 6**

**Description:** Programmable autorotation flight of dandelion-inspired microfliers.

**File Name: Supplementary Movie 7**

**Description:** The light-fueled mid-air flight of a real dandelion seed.
